# Using community participation to assess acceptability of “*Contra Caries*”, a theory-based, *promotora*-led oral health education program for rural Latino parents: a mixed methods study

**DOI:** 10.1186/s12903-015-0089-4

**Published:** 2015-09-03

**Authors:** Kristin S. Hoeft, Sarah M. Rios, Estela Pantoja Guzman, Judith C. Barker

**Affiliations:** Department of Epidemiology and Biostatistics, Center to Address Disparities in Children’s Oral Health (CAN DO), 3333 California Street, Suite 485, San Francisco, CA 94143 USA; Department of Anthropology, History & Social Medicine, Center to Address Children’s Oral health Disparities (CAN DO), University of California San Francisco, 3333 California Street, Suite 485, San Francisco, CA 94143-0850 USA

## Abstract

**Background:**

Latino children experience more prevalent and severe tooth decay than non-Hispanic white and non-Hispanic black children. Few theory-based, evaluated and culturally appropriate interventions target parents of this vulnerable population. To fill this gap, the *Contra Caries* Oral Health Education Program, a theory-based, *promotora*-led education program for low-income, Spanish-speaking parents of children aged 1–5 years, was developed. This article describes qualitative findings of the acceptability of curriculum content and activities, presents the process of refinement of the curriculum through engaging the target population and *promotoras*, and presents results from the evaluation assessing the acceptability of the curriculum once implemented.

**Methods:**

Focus groups were conducted with low-income Spanish-speaking parents of children 1–5 years living in a city in an agricultural area of California. Interviews were digitally recorded, translated and transcribed, checked for accuracy and the resulting data was thematically coded and analyzed using a social constructionist approach. The *Contra Caries* Oral Health Education Program was then implemented with a separate but similar sample, and after completing the program, participants were administered surveys asking about acceptability and favorite activities of the education program. Data were entered into a database, checked for accuracy, open-ended questions were categorized, and responses to close-ended questions counted.

**Results:**

Twelve focus groups were conducted (*N* = 51), 105 parents attended the *Contra Caries* Oral Health Education Program, and 83 parents filled out surveys. Complete attendance and retention was high (89 % and 90 %, respectively). This study found that their children’s oral health is a high priority. Parents were not only interested in, but actually attended classes focused on increasing their knowledge and skills with respect to early childhood oral health. The *Contra Caries* content and format was perceived as acceptable by parents. Strong opinions about curriculum content were expressed for including information on how caries starts and progresses, weaning from the bottle, oral health care for children and adults, motivational strategies for children’s tooth brushing, dental visits and cavity restorations.

**Conclusions:**

The *Contra Caries* Oral Health Education Program was acceptable to low-income, Spanish-speaking parents of children 1–5 years. Participating in the curriculum development and revision process likely played an important role in the parents’ high acceptability of the program.

## Background

Early childhood caries (ECC), or tooth decay of the primary dentition, affects 28 % of children aged 2–5 years in the United States [1]. ECC causes pain that can interrupt activities of daily living such as eating, sleeping, playing and talking, and can result in serious health consequences if left untreated. Pain resulting from ECC and the time to seek treatment can result in missed work for parents, missed school for children, and reduced school performance [2]. In addition, ECC can have long-lasting effects on oral health, self-esteem, and even employment [3, 4]. ECC is a multifactorial and largely preventable disease, with influences at multiple levels of organization, from individual to societal [5].

Mexican American children are more likely than non-Hispanic white and non-Hispanic black children to have experienced ECC, have more affected teeth, more severely affected teeth, and more untreated decay [6, 7]. Rural, migrant and farm-worker children have especially poor oral health, particularly if they are also Latino [8, 9]. The Latino population is the fastest growing and largest minority population in California and the U.S. Overall, 38 % of California’s population is Latino and of these over 14 million people, 83 % are of Mexican-origin [10, 11].

While it isn’t known exactly why Latino children experience disproportionately high prevalence and severity of ECC compared to non-Hispanic white and black children, initial research suggests that there are multiple inequalities experienced by this population including language and health literacy, access to and perceived need for oral care, rejection of tap water consumption, sugary drink and juice consumption, confusion around infant bottle use and late bottle weaning [3, 12–18].

While not the only step in prevention, oral health education is a critical and important factor in preventing ECC. Education is especially important for this vulnerable population, since many Latinos have less access to other proven ECC interventions, such as professional oral health care [14]. Theory-based interventions are more successful than those with other approaches [19], and education designed with and for particular cultural and linguistic groups are more successful than those developed generically and simply translated for other target populations. Using *promotora*, i.e., trained lay health educators from the local community, is a culturally appropriate approach to delivery of health interventions, and have been shown to be effective at creating behavior change for other (non-dental) health conditions [20–23].

However, most existing ECC prevention education programs lack a theoretical basis and have not been formally evaluated. They have not involved *promotoras* and are focused at preschool age and older children directly, thus missing the critical prevention window for children under age 3 who are primarily dependent on their parents or caregivers for access to dental visits, exposure to and development of home care habits (such as providing the child with a toothbrush and toothpaste, tooth brushing assistance), as well as diet (for example, low-cariogenic foods and drinks). Finally, very few education programs are developed for specific vulnerable populations such as Latinos.

Given the high need for oral health promotion education in low-income, rural, Latino populations in California, we set out to develop and test *Contra Caries* Oral Health Education Program (referred to as *Contra Caries*), a theory-based, *promotora*-led oral health education program targeting low-income, Spanish-speaking parents of children aged 1–5 years. A preliminary curriculum consisting of four sessions on key topics was developed in Spanish by the authors based on: 1) findings in the literature from previous ethnographic research in similar Latino populations [3, 18, 24] which helped identify knowledge, behaviors, and skills to target with the intervention; and 2) two specific theories. First, we employed Stokols’ social ecological model as a broad contextual framework within which we adapted Bandura’s Social Cognitive Theory [25, 26]. Stokols describes behavior influences at five levels: intrapersonal, interpersonal, organizational, environmental, and socio-cultural. While *Contra Caries* was too small to examine influences at all five levels, it was designed to be mindful of the other influences upon an individual’s behavior and provide suggestions and support for counteracting barriers encountered at the more structural rather than familial levels. Social Cognitive Theory is a comprehensive social-psychological theory which is compatible with a social ecological approach and includes key constructs (such as self-efficacy) known to positively affect oral health and influence daily behavior [27]. Bandura’s model lends itself to not just increasing parental oral health knowledge but also to skill-building and behavior change.

### Objective

This article describes qualitative findings of the initial acceptability of curriculum content and activities, presents the process of refinement of the curriculum through engaging the target population and *promotoras*, and presents results from the evaluation assessing the acceptability of the curriculum once implemented.

## Methods

**Fig. 1:**
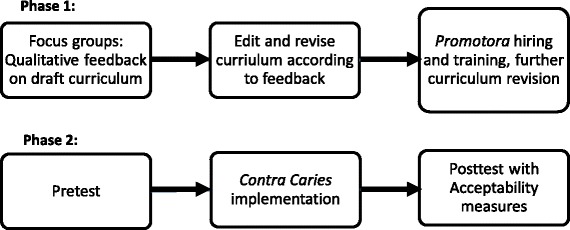
Steps undertaken in each of the two study phases

This study was conducted in a medium-sized city in an agricultural area of California. Because the city’s economy is so heavily based around the production and distribution of agricultural products and associated services, we use the phrase ‘rural’ in this manuscript to distinguish it from urban sites with more diversified economies. Because rural Latino children have very poor oral health [1,3,4,6,8,9], we chose to locate this study in a rural setting, in a different geographic location than our previous ethnographic work, but with a similar Latino immigrant population. Figure 1 presents in schematic form the steps undertaken in the two phases of this project, with the methods of each detailed separately below.

### Phase 1: focus groups

We partnered with Community Oral Health Services, a community-based organization that focuses on providing dental services to underserved populations, co-locating in their office that is well situated in a predominantly Latino neighborhood of the community. This organization facilitated introductions to other community organizations for participant recruitment.

Phase 1 data collection consisted of focus groups with community members so they could assess the draft content, format, and logistics of the draft curriculum [28]. Qualitative focus groups were conducted in Spanish and led by two bilingual researchers. Proposed class activities integral to the *Contra Caries* curriculum were presented to the focus groups participants who were asked to give detailed feedback on what they liked, didn’t like, and how they would like to change things, both for content of the lessons and the format of the activities. Focus group inclusion (selection) criteria required participants be low-income, Spanish-speaking parents or caregivers of at least one child between ages 1 and 5 years. Participants were non-randomly recruited (convenience sample) from community festivals and the Special Supplemental Nutrition Program for Women, Infants, and Children (WIC). Focus groups lasted 2 hours, were held in community organization sites, and participants received a $25 gift card to a local supermarket for participation. Focus groups were conducted until we reached saturation on the key topics of interest. Discussion was recorded and the audiotapes transcribed verbatim, checked for accuracy, and observations of interaction during the groups were summarized as typed field notes. The resulting data was thematically coded and analyzed using a social constructionist approach, developing codes from the data [29, 30] in an inductive process, and using QSR International’s NVivo 10 software to apply codes to transcript text [31]. All study procedures were reviewed and approved beforehand by the Committee for Human Research (Institutional Review Board) at the University of California, San Francisco (Approval number 11–05603). The study was undertaken with the understanding and written consent of each parent/caregiver participant prior to participation.

The information from the focus groups was used to revise the curriculum content and delivery style. Then *promotoras* were hired and trained in order to conduct a pilot study of the full curriculum. Potential *promotoras* were recruited through flyers and in-person recruiting in local businesses (such as *tiendas* and laundromats) and social services such as WIC. A bilingual application form was given to interested applicants, applicants were interviewed in Spanish, and 4 of the 14 applicants were selected and hired as *promotoras*, based on the criteria identified in the focus groups (reported in Results below). Training of the *promotoras* was conducted intermittently over a 5 month period, ranging from a few hour orientations 3 days a month at the beginning, to all-day practice sessions two days a week by the end. Training included teaching oral health information using the *Contra Caries* curriculum, as well as group facilitation, study procedures and record keeping, and ethics. *Contra Caries* curriculum was even further refined with input from *promotoras* as they practiced leading the curriculum in preparation for its implementation.

### Phase 2

Phase 2 involved implementation of the revised *Contra Caries* classes and use of surveys asking about acceptability and favorite activities of the education program. A separate, larger convenience sample of participants was recruited with the same inclusion criteria, recruitment procedures, and consent process as in Phase 1. These participants signed up to attend *Contra Caries* classes, 4 sessions of two-hours each, and to fill out surveys. Classes were held in community locations such as WIC classrooms or apartment building common rooms, and were led by the trained *promotoras*.

A brief overview of curriculum content is provided in Table 1. A number of teaching modalities were employed in delivering the oral health information to study participants, including group discussion and sharing, *promotora* demonstrations, interactive group problem-solving, visual story-telling, goal setting and check-ins, practicing skills, and giving and receiving feedback. Activities incorporated throughout the sessions were designed to reinforce topical content: for example, spooning out sugar to visualize the quantities on nutrition labels, using plaque disclosing tablets (that temporarily dye areas of dental plaque) to reveal toothbrushing effectiveness, walking through the typical daily activities of a toddler in visual story form and demonstrating how each activity relates to caries formation.

**Table 1:** Overview of final curriculum topics

Class session	Summary of topics covered
Session 1	Introduction, goal setting, description and importance of baby teeth, process of cavity formation and overview of cavity prevention
Session 2	Details of how brushing with fluoride toothpaste helps prevent cavities, current tooth brushing technique, use of plaque disclosing tablets, demonstration and practice of ideal tooth brushing technique, demonstration and practice of dental floss technique, specific techniques for brushing children’s teeth, lift-the-lip exam, behavior management and motivation for brushing children’s teeth
Session 3	Details of the role of diet (types of food/drink, frequency, and bottle/sippy cup use) in causing and preventing cavities, how to transition away from the bottle/sippy cup, how to identify sugar in foods (nutrition labels) and healthy snack foods
Session 4	Details of the role of professional dental care in prevention and treatment of cavities, process of making appointments and attending dental checkups, overview of dental treatments (from prevention through restorations), dental behavior management techniques, making children feel comfortable at the dental visit, local resources, review game, certificates of course completion

Study participants received $5 per class attended, and $20 per survey for completing the baseline and immediate post-intervention survey and $30 for completing a 3-month post-intervention survey as compensation for their time. Results presented in this report are about acceptability only; 3-month post-intervention data and their comparison with baseline or immediate post-test data about knowledge and practices are reported elsewhere. Questions in the pretest survey included demographic information, whereas questions in the posttest immediately after attendance at the fourth and final class asked participants about the degree to which they liked the classes using a five point Likert scale, and their favorite and least favorite activities using open ended questions. Surveys were administered in-person by Spanish-speaking research staff different from those involved in giving the classes. Open-ended questions were categorized, and responses to close-ended questions counted. Data were entered into a Microsoft Access database, with 10 % of the data being double-entered by different researchers and checked for accuracy, achieving 100 % agreement. Descriptive statistics were conducted using Stata 13.0 [32]. Attendance data was recorded by participants themselves on a sign-in sheet, and checked and revised by the *promotoras*.

Presented in the Results section are findings from the Phase 1 focus groups on acceptability of oral health education generally, acceptability of curriculum content, preferred activities and lesson format. Phase 2 results present survey data of acceptability of the fully implemented *Contra Caries* program. Participant quotes illustrate typical themes and comments in the data. When multiple speakers converse back and forth, interviewers and each respondent are noted using < >.

## Results

### Phase 1

Twelve focus groups were conducted (*N* = 51). Participants were primarily mothers, though a few grandmothers, aunts, and one foster parent also participated. Participants were low-income, with on average a less than high school education (Table 2). The mean age of the child aged between 1–5 years, the focus of the education intervention, was 3 ± 1.5 years; and 15 % of children were parent-reported as having never been to the dentist. All lived in the rural community in which the study was conducted.

**Table 2:** Demographics for the Spanish-speaking caregivers in Phase 1 focus groups (*N* = 51)

	% or mean (±SD)	Range
Caregiver age (years)	31.4 ± 9	20-60
Caregiver is mother	88 %	
Family size	4.3 ± 1.9	1-8
Annual family income^a^	$19,000 ± 9,400	$5,760-50,000
		Median = $16,800
Years education	8.9 ± 3.9	1-17
Born outside United States	90 %	

^a^ *n* = 29 due to missing data, either “don’t know” or skipped question

Major results from the community focus groups that influenced the final content and format of the *Contra Caries* curriculum encompassed acceptability of: learning about children’s oral health, *promotoras* as class leaders, curriculum content, and activities and lesson format.

### Acceptability of learning about children’s oral health

Participants were interested in learning about oral health for their children. Many had children with caries experience and were interested in preventing future tooth decay as well as understanding more about their previous dental treatment experiences. A common desire expressed by most participants was the importance of opportunities, such as education, available here in the United States compared to their life situation before immigrating. They were very interested and committed to provide improved opportunities for their children, as this mother explains:

“…It’s very interesting for one to look at the progress of one’s children and knowing that they are creating a better life for themselves than the one we’ve had. They have a lot of opportunities now to live a healthier life, not just in oral health, but physical health and everything, because now they have a lot of knowledge that we… well, I can say in my case, I didn’t have. And yes, I lacked that, but now I give thanks to God for these new opportunities that we have to learn and to instruct our children to learn what we didn’t have.”

To make classes work for parents’ busy lives, they wanted classes in their neighborhoods in familiar and convenient locations, and for them to be conducted in Spanish. Stay-at-home parents preferred weekday mornings when older children were in school while working parents preferred evening or Saturday times. Providing free childcare during the classes was absolutely necessary for parents to be able to attend.

### Acceptability of *promotoras*

Participants wanted *promotoras* who have “a good character and that you feel enough trust to express your point of view.” Participants preferred classes be taught in Spanish by women, particularly mothers who have experience raising and caring for children. These views were expressed in several focus group sessions:

<respondent2> I don’t want to be a feminist, but I’d prefer a woman because women always have that thing of being a mother… more caring.

<respondent1> It’s not just that, I think that we mothers are the ones that are on top of that, telling our children to brush their teeth… they have the experience. As a mother you are the one that makes them do more things than the fathers.

…

<respondent1> And they can give us their experiences that she had with her children, how she taught them and all that.

### Acceptability of activities and lesson format

Participants preferred group class format where they could learn from each other, share their thoughts, and learn about experiences and thoughts of other caregivers. They strongly preferred the education intervention to be given using the Spanish language, and liked pictures, illustrations and diagrams over written text. They preferred an interactive, rather than didactic educational format:

<respondent1> When a teacher is saying okay, and showing you the bulletin, ‘do this, do this, do this, do this,’ that doesn’t work. You aren’t going to catch everything in the same way compared to being in a group and we’re saying ‘okay, hold their mouth… this is this,’ and going over it so you-

<respondent3> Where you’d have more visual things.

<respondent1> Yes.

<respondent2> You are mentally catching it all because you are doing it and if you are just listening, your mind wanders.

<respondent3> Yes.

<respondent2> [thinking about] ‘I need to make dinner.’

<respondent3> That’s why I think it’s best for it to be more visual and entertaining to do.

Participants were less interested in written brainstorming or other writing activities:

*<interviewer1> And do you like to write your ideas or do you prefer to talk about them and not write?*

<respondent2> Well, I think it’s better to talk because when you write you forget things, like what happened to me, and it’s best to talk about them… <respondent4> Yes, because at that moment our thoughts are coming to us and when you write you don’t remember and think “what else can I put, what else can I put?”

<respondent2> Yes, it’s best to just talk about it.

All of these suggestions were integrated into the revised, final curriculum. The content and sequencing of topics in the *Contra Caries* curriculum delivered during the pilot study appears above in Table 1.

### Acceptability of curriculum content

As shown in Table 1, the draft curriculum proposed to cover topics such as the role of bacteria in caries and caries etiology, tooth eruption, tooth brushing for children, tooth brushing and flossing for adults, nutrition, using and stopping use of the bottle for infants, dental visits and cavity restorations. We asked caregivers what topics they would like to learn in class, and then to rank all the topics by priority. Particular topics around which participants had very strong opinions and engaged in considerable discussion helped to shape the final curriculum. This included information on how caries starts and progresses, weaning from the bottle, oral health care for adults, motivational strategies for children’s tooth brushing, dental visits and dental treatments and restraints.

### How caries starts and progresses

We asked parents whether we should include what caries is, the role of bacteria, and how the bacteria and disease progress with repeated exposure to carbohydrates/sugar along with lack of fluoride and poor hygiene practices. Parents felt this was an extremely important foundation for the rest of the classes and that this information needed to be presented first so that everything else could relate to this foundation. They helped develop a narrative thread, an analogy to which they related – that of protecting your house [teeth/mouth] from ants [bacteria] -- as a way to integrate the various topics, and to ensure the language of the lesson used familiar words and concepts and remained coherent and relevant to a low literacy audience.

### Children using the baby bottle

Parents were unreceptive to suggestions based on the professional dental and pediatric literature of transitioning children from drinking from a baby bottle to a cup at 12–18 months old, the recommended age [33]. They felt 12 months old was too young for a child to stop using a bottle and that 18–24 months was a more acceptable age. This excerpt from a focus group discussion illustrates this point:

*<interviewer> Do you think [stopping the bottle] would work for children who are one year old?*

<respondent1> It might be very early for them.

<respondent3> They would be very young. They don’t understand yet.

<respondent1> Maybe a year and a half.

*<interviewer2> A year and half?*

<respondent1> I think so, because at that age [one year] they are very young, right?

<respondent3> Yes, a lot of the time when they are a year old and you want to talk to them, they don’t pay attention to you, they don’t understand you. So, you are going to be fighting with them over the bottle and they will want the bottle and they won’t understand.

<respondent1> They are just starting to walk.

<respondent3> Yes. And you have to give them the bottle because the child won’t understand and they are going to continue to cry and get desperate for their bottle and you’ll say “be quiet”. Yes, because I had that struggle with them. When they are older they get off it, but when they are a year old, that’s very young.

Even with discussions of transitional techniques such as giving bedtime bottles with water, or cups of milk separate from bedtime, parents felt very strongly that children were not developmentally ready to stop drinking from a baby bottle at 12–18 months of age.

### Oral hygiene for adults

Another topic area we debated including was oral hygiene for adults because the objective of the education intervention was for improving children’s oral health. Parents, however, really liked learning how best to brush and floss their own teeth. For many, this was the first instruction on flossing they had ever had. They felt it was important for their health as well as for their ability to set a good example for their children to learn how to properly care for their own teeth. And once they learned about how caries-causing bacteria can be transmitted between family members, they felt even more strongly that the instruction on adult hygiene had to stay in the curriculum. They liked the hands-on approach and in-class practice that the curriculum included, such as *promotora* demonstrations with large props depicting teeth, gums and brushing activity, and practicing in front of a mirror. They were comfortable using plaque disclosing tablets in a group setting. A few pregnant mothers chose not to participate in the disclosing tablet activity, due to nausea and uncertainty around the possible effect of the disclosing tablets.

### Motivational strategies for children’s tooth brushing

Participants expressed difficulty with overcoming the resistance of fussy children and with motivating their children to brush their teeth, because

“it’s difficult for them because, for one, you have to be telling them and a lot of the time with the breakfast, the house, the chores, or whatever, then you can’t be on top of everything”

and

“because it’s difficult for kids, especially when they don’t want to brush them at night… ‘I’m sleepy! I want to go to bed!’ So, that’s when you do your job as a parent, as a mother.”

Parents were very interested in techniques, strategies, and activities they could do with their children to improve tooth brushing cooperation. This was viewed as being especially important once parents learned the proper techniques and time required for optimal brushing. They shared and discussed many suggestions of successful techniques they themselves had used, as well as examples of struggles and failed motivational techniques. Favorite successful techniques were songs, taking turns, parent or sibling modeling, letting children select toothbrush or toothpaste with cartoons, letting children play with the water or toothbrush after brushing, and praise.

### Dental visits

Despite most participants having children with dental restorations, few received (or remembered getting) explanations from the child’s dentist at that time. As these caregivers explained, “because they [dentists] don’t really explain well about what they are going to do, how they are going to do it and the consequences that there are going to be.” Caregivers wanted to learn about their rights at the dental office, and when it is acceptable to ask questions and for more information. This discussion illustrates these points:

<respondent 4> [I want to learn about] the dental office. I understood that we have the right to get information or ask them [questions] and that they answer what we want to know about the treatments and about things… about everything to do with our children’s dental care, to have information that we would like to know…

*<interviewer2> Good. Okay.*

<respondent4> There are places that have people who don’t want to help us or give us information.

<respondent3> And you are left with questions.

<respondent4> Yes. And I think that when it comes to everything about doctors and all that, I think that they should have that… give us information or answer our questions about why we are there.

### Dental treatments and restraints

Parents wanted very detailed information not just about what exactly was happening during the decay process but also what the corresponding dental treatment was. They wanted, for example, to know not just formal terms but exactly what a filling is, how it differs from a crown, and when a child might need one or the other, as explained here:

<respondent5> When to know when a filling or crown is necessary.

*<interviewer2> Okay. Good.*

<respondent2> When is it okay to put those teeth, those ones they put when they have cavities, the silver ones at the front, are they silver?

*< interviewer2> When is it necessary, or at what age?*

<respondent3> When they need to be put.

<respondent2> … what age they should put those on their teeth.

*<interviewer2> Cap them?*

<respondent2> Yes, because they put those on my son when he was two. He was very young and they didn’t explain what exactly they were going to do. When it came out that they had done that, he came out with marks from having his mouth open… marks on his face. We thought it was something more simple, but it wasn’t.

<respondent3> They didn’t explain what they were going to do?

<respondent2> No.

Participants also wanted a lot of detail around behavior management techniques some dentists use, such as what they called “tying down” (i.e., use of a “papoose board” or other physical restraint) and sedation. These were areas of high anxiety for caregivers. Because many times parents were not allowed into the treatment rooms during their children’s dental treatments, they wanted to know what was happening to their children in the dental operatory, as this excerpt from the focus group explains:

“He was about four years old when they had to pull out his two front teeth, and they didn’t explain that to me either. He came out… with a lot of marks, but too much… on his face, and on his arms, maybe because it was tight, but they didn’t explain that. They just said that there was going to be some anesthesia and that he would be slightly drowsy, but that’s it. And they didn’t tell me that I couldn’t be there either. I wanted to go in but they told me that I couldn’t be there. They never told me that I couldn’t be there and that they were going to tie him up so that he doesn’t move, or that they were going to put something on him.”

### Phase 2: Post-test survey acceptability questions

The implementation of the *Contra Caries* Oral Health Education Program involved 105 participants. They were mainly Mexican-born mothers and about half had graduated high school (Table 3).

**Table 3:** Self-reported demographic characteristics of low-income Spanish-speaking parent or caregiver and their child closest to 3 years in Phase 2, delivery of the educational intervention (*N* = 105)

Caregiver characteristic	Count (%) or mean ± SD; median; range
Mothers	81 (77 %)
Caregiver birth country	
U.S.	11 (10 %)
Mexico	91 (87 %)
Age (years)	33.7 ± 8; median = 33; range = 18-57
Years completed in school	
6 years or less	35 (33 %)
7–11 years	18 (17 %)
High school diploma	33 (31 %)
More than high school	19 (20 %)
Number of children	2.4 ± 1.1; median = 2; range = 0-5
Child characteristic	
Female	47 (45 %)
U.S. born	102 (98 %)
Never had dental visit	14 (13 %)
Age (years)	3.0 ± 1.3; median=3; range=0-5

A total of 105 participants attended the *Contra Caries* Educational Program, with 83 completing the posttest survey with acceptability questions immediately after the last of the 4 educational sessions (12 other people (95 total) filled out the posttest survey, but the first version did not include acceptability questions). Thirteen classes were held, with between 5 and 14 students in each class (mean 7.7). Each session took two hours, with classes held on weekdays, mainly in the mornings and a few in late afternoons. Despite asking people, especially parents with young children, to commit to attend a class at a set day and time for four weeks in a row, attrition was low. Overall, the retention rate was 90 %, with 89 % of retained participants attending all 4 sessions, and only 5 people missing more than 1 session. Reasons for missed sessions were illness of participant or one of their children, or work schedule conflicts. Most people who missed a session arranged to attend other classes to make up sessions. A strong majority (95 %) of respondents said they liked the class very much, 2 (2 %) participants said they liked it a little, and 2 (2 %) said “so-so”. No participants reported not liking it very much, or not liking it at all.

Participants were asked an open-ended question about their favorite class activity, allowing compound answers. The most commonly mentioned favorite class activity was learning the specific steps to brushing teeth, with about half of participants (53 %) listing tooth brushing generally, or a specific aspect of tooth brushing instruction such as how long to brush for, or how to hold or move the toothbrush. Six parents specifically valued aspects of instruction related to managing children, such as using dolls as learning models, or tactics to motivate children’s interest and cooperation in brushing (e.g., singing). The next-most popular class activities were flossing (16 %), all activities (16 %), a review game covering all topics (11 %), sugar and nutrition labels (10 %), and decay process (9 %).

Open-ended questions were also asked about class format. Class size was a common topic of both satisfaction and dissatisfaction (some wanted larger class sizes more like school classes, and others wanting smaller more intimate sizes). But the familiar, convenient location and time of class were listed often as things liked about the class, as was the sharing and social nature of the activities, and the use of *promotoras* as the educators.

## Discussion

This study found that their children’s oral health is a high priority for low-income Spanish-speaking parents. They were not only interested in, but actually regularly attended classes for parents focused on increasing their knowledge and skills with respect to early childhood oral health. *Contra Caries* Oral Health Education Program content and format was perceived as highly acceptable.

Very little has been written about the acceptability of various children’s oral health educational interventions, including those aimed at Latino families. Some research examined acceptability of various ECC prevention methods, including tooth brushing with fluoride toothpaste, and found it acceptable to parents [34], but a formal educational platform of how to guide parents to conduct those prevention methods was not examined. Despite this lack of acceptability literature in oral health education specifically, *promotora*-based education is well-received in similar populations on other health-focused topics [20–23]. While some *promotora* programs have been developed around oral health [35, 36], to our knowledge they have not previously been evaluated for acceptability.

Parents reported liking a group class setting with other parents, and preferred to be taught by lay people (*promotoras*) who had children over health education or dental professionals, consistent with reports of *promotora* interventions being acceptable to this population [37]. Similar to previous research with the Latino population that has reported low knowledge about the role of bacteria and sweet liquids in baby bottles as contributing factors to ECC [3, 18], prevention topics of particular importance to parents in this study included information on how caries forms and progresses, oral health care for adults, motivational strategies for encouraging children’s tooth brushing or overcoming their resistance to this activity, issues that again coincide with previous research findings with similar populations [24]. Evidence-based motivational strategies around tooth brushing techniques for children are not currently in the literature; current approaches will need further research. However, research demonstrating that maternal self-efficacy has a role in children’s oral hygiene practices supports the idea that having specific strategies that increase parents’ confidence to carry out children’s oral hygiene could be beneficial [27].

The desire for parents to receive information about adult dental health and details of dental treatment procedures, however, was new information not reported in previous research. This was reassuring since the literature on vertical transmission of cariogenic bacteria supports the inclusion of adult oral health education as a component in child-focused oral health interventions [38]. This desire for education to apply to all family members is not surprising, given the well-documented cultural value of familism in the Latino population [39].

Parents’ strong desire to learn very detailed information about dental visits and cavity restorations is not presently noted in the literature. Differences in dental care between (migrant) parents’ home country and the U.S., low utilization rates (and thus familiarity) with dental care for parents, language and health literacy barriers, and high dental need of their children resulting in extensive behavior management and advanced restoration techniques, could all be contributing factors to this expressed desire. In addition, very little data is available on acceptability of oral health behavior management techniques, such as use of restraints or sedation, in the Latino population specifically, despite the significant caries burden in this population.

The issue of parents not agreeing with the professional guidelines that children be weaned from using the bottle at 12–18 months of age is consistent with literature reporting later weaning in this population [40]. However, given the strong contribution of prolonged bottle use to dental caries, it is an area that warrants further, collaborative research to identify facilitators and barriers to timely weaning, and interventions or tools to equip and motivate parents to successfully wean their young children. Some preliminary comparative research identified early introduction of a cup and trust/communication with health providers as some characteristics of Latino families who were able to wean their children from the bottle at an earlier age [41].

Finally, survey results after participation in the *Contra Caries* program reinforce initial findings from the focus groups—this topic and format is of interest to parents, and 95 % of participants enjoyed the *Contra Caries* program “very much”. Overall, 90 % of participants who started the program continued attending.

Like all research, this study has limitations. Social desirability bias is definitely a potential factor to consider, especially with survey answers after the intervention. Participants may have been hesitant to criticize the program, although we attempted to mitigate this by having research staff and not the *promotoras* from the classes administer the survey, and having researchers emphasizing an interest in receiving negative feedback. Both phases of this project used a convenience sample recruited from community services and housing, who may have been more motivated, more connected to services, or different from the general population in important ways. These samples, however, matched well samples reported in other studies of low income Spanish-speaking parents and their views on children’s oral health, which suggests the sample was reasonably representative of this population sub-group [3,8] We made attempts to minimize potential bias from recruitment limitations through reaching out to a variety of community settings, for example in Phase 2 both daytime recruitment to apartment complexes to reach stay-at-home parents, as well as recruitment from a daycare center which serves working parents. Whatever bias remained in our recruitment strategy limits the generalizability of our results, thus we caution readers that care should be taken when generalizing to Latinos with another socio-economic status, geographic location, or migration history. Finally, participants were offered compensation for the time they spent participating in the intervention and research procedures. It is possible that this compensation inflated attendance rates, although we limited the payment per class attendance to only $5.00 per class to attempt to minimize this. Future, larger and randomized studies of the curriculum would be desirable in order to test efficacy of the curriculum at improving oral health related knowledge and behaviors, as well as the cost and benefit of widespread implementation of the program.

## Conclusion

Latino parents are interested and motivated to learn about improving their children’s oral health. Important topics include how caries starts and progresses, adult and children’s oral hygiene including motivational techniques for children, and dental treatments and restraints. The *Contra Caries* Oral Health Education Program was acceptable to low-income, Spanish-speaking parents of children 1–5 years and attendance, retention, and acceptability of the program were high. Participating in the curriculum development and revision process likely played an important role in the parents’ high acceptability of the program. Additional research into efficacy and cost-effectiveness of the program is warranted.

## Abbreviations

ECC: Early childhood caries
WIC: Special Supplemental Nutrition Program for Women, Infants, and Children
